# Treatment patterns and clinical outcomes for patients with melanoma and central nervous system metastases: A real‐world study

**DOI:** 10.1002/cam4.4438

**Published:** 2021-12-07

**Authors:** Hussein Tawbi, Tu My To, Karen Bartley, Natalia Sadetsky, Elizabeth Burton, Lauren Haydu, Edward McKenna

**Affiliations:** ^1^ Department of Melanoma Medical Oncology The University of Texas MD Anderson Cancer Center Houston Texas USA; ^2^ Genentech, Inc. South San Francisco California USA; ^3^ Department of Surgical Oncology The University of Texas MD Anderson Cancer Center Houston Texas USA; ^4^ Present address: Seagen Bothell Washington USA; ^5^ Present address: Atara Biotherapeutics South San Francisco California USA

**Keywords:** CNS metastases, melanoma, overall survival, radiotherapy, systemic treatment

## Abstract

**Background:**

Patients with melanoma and central nervous system (CNS) metastases have poor survival outcomes. We investigated real‐world treatment patterns and overall survival (OS) of patients with melanoma and CNS metastases.

**Methods:**

A retrospective analysis utilizing a nationwide de‐identified electronic health record‐derived database was undertaken in patients diagnosed with advanced melanoma between January 2011 and September 2018. Patients with any visit ≤90 days of metastatic diagnosis and with confirmed CNS metastases were included.

**Results:**

Of 3473 patients diagnosed with advanced melanoma, 791 patients with confirmed CNS metastases were identified and included in this analysis. Synchronous CNS metastasis (≤30 days of metastatic diagnosis) was associated with longer median OS than metachronous CNS metastasis (>30 days after metastatic diagnosis, 0.58 vs 0.42 years). Stereotactic radiosurgery (SRS) was the most common treatment (40.5%) alone or in combination with other local or systemic therapies, being more frequent in patients diagnosed in 2015+ versus 2011–2014 (44.1% vs 35.5%, respectively). The most common systemic treatment was immune checkpoint inhibitors (ICIs; 30.5%), predominantly anti‐cytotoxic T‐lymphocyte antigen 4 (CTLA‐4) alone (2011–2014) and anti‐programmed death‐1 alone or in combination with anti–CTLA‐4 (2015+). Median OS was longest in SRS‐treated patients (1.17 years) regardless of number of CNS metastases. Median OS for SRS‐treated patients increased from 0.83 years (2011–2014) to 1.75 years (2015+). In multivariable analysis, the effect of SRS remained significant after adjustment for sex, race, intracranial and extracranial disease burden, and timing of CNS metastases. Interaction testing to examine potential synergy between SRS/whole‐brain radiation therapy and ICIs found no significant interaction.

**Conclusions:**

Despite advances in treatment, patients with melanoma and CNS metastases have poor survival outcomes. Prevalence of SRS increased over time and was associated with improved outcomes.

## Graphical abstract

This retrospective analysis of real‐world data demonstrated that patients with melanoma and CNS metastases have poor survival outcomes despite recent advances in treatment options. Prevalence of stereotactic radiosurgery increased over time and was associated with improved outcomes.

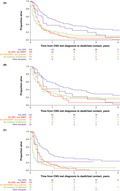

## 1 INTRODUCTION

Among common solid tumors, melanoma has the highest risk of metastasis to the central nervous system (CNS).^1^, ^2^ Up to 75% of patients with metastatic melanoma will develop CNS metastases during their disease,^3^ most within 3 years of melanoma diagnosis.^1^ CNS involvement adversely affects quality of life and is historically associated with poor prognosis, with median overall survival (OS) of 4–6 months in patients with melanoma with CNS metastases.^1^, ^3^, ^4^

Standard treatments for melanoma with CNS metastasis include whole‐brain radiation therapy (WBRT) and stereotactic radiosurgery (SRS), alone or in combination, and cytotoxic chemotherapy.^5^ Unfortunately, these treatments have not shown significant improvement in OS. New treatments approved from 2011 to 2015, including BRAF inhibitors (BRAFi)^6^, ^7^ and MEK inhibitors (MEKi)^8^, ^9^, ^10^, ^11^ for *BRAF* ^V600^ mutation‐positive tumors and immune checkpoint inhibitors (ICIs)^12^, ^13^, ^14^ have improved treatment outcomes for some patients. Patients with unfavorable prognostic factors, including CNS metastases and elevated lactate dehydrogenase levels, often experience less benefit.^15^, ^16^, ^17^ Furthermore, patients with CNS metastases have largely been excluded from registrational studies of novel treatments for melanoma. Thus, there remains substantial unmet need for treatment options that improve OS in this patient population.

Immune checkpoint inhibitors^18^, ^19^ and BRAF/MEK inhibitors^20^, ^21^, ^22^, ^23^ have demonstrated intracranial activity. This activity is of limited duration (BRAFi ± MEKi)^21^ or is associated with a high rate of toxicities despite durable benefit (ICIs),^18^, ^19^, ^24^ suggesting the need for alternative or combination treatments. Real‐world data on treatment patterns and outcomes can inform treatment options and identify groups with high unmet needs, but data are lacking in the context of newer treatments. The objective of this study was to characterize real‐world treatment patterns and survival of patients with melanoma and CNS metastases in the United States.

## 2 METHODS

### 2.1 Patients

This retrospective study utilized data from the Flatiron Health database (Flatiron, NY, NY), a nationwide longitudinal, de‐identified database derived from electronic health record data.^25^ During the study period, the de‐identified data originated from ~280 cancer clinics (~800 sites of care). The patient cohort for this study comprised a random sample of eligible patients selected for enhanced manual chart review. De‐identified patient‐level data included structured and unstructured data, curated via technology‐enabled abstraction.^26^ Institutional review board approval of the study protocol with a waiver of informed consent was obtained prior to study conduct.

Patients diagnosed with advanced melanoma from January 2011 through September 2018 (inclusive) were eligible for selection. Patients with melanoma were selected by International Classification of Diseases (Ninth and Tenth Revision) codes ICD‐9 172.x or ICD‐10 C43x or D03x. Patients with CNS metastases were selected and confirmed through review of clinical or pathologic records. Extracranial disease burden was captured by distinct anatomic sites and confirmed through review of medical records and pathologic reports. Synchronous versus metachronous CNS metastasis was defined using a cut‐off of 30 days after first metastatic diagnosis to account for variability in local practice and access to care.

Patients with any visit with the Flatiron Health network ≤90 days of metastatic diagnosis (metastasis in any region) and confirmed to have CNS metastases at first metastatic diagnosis or at any time during follow‐up were included. Of 3473 patients diagnosed with advanced melanoma, 791 patients who had a metastatic diagnosis date, a visit ≤90 days of metastatic diagnosis, and a CNS metastatic diagnosis were included in the current analyses (Figure S1).

### 2.2 Treatment

Initial treatment for CNS metastatic disease was defined as first therapy (local and systemic) started ≤90 days of CNS metastasis diagnosis date and any other therapies received ≤90 days of that first therapy. Treatments were classified as systemic (i.e., immunotherapy, targeted therapy, chemotherapy, other nonlocal therapies) or local (WBRT, SRS, other radiation therapy, craniotomy/metastasectomy). Systemic therapies were further categorized by therapeutic class. ICIs included anti‐programmed death‐1 (PD‐1) or anti‐programmed death ligand‐1 (PD‐L1) therapies (i.e., pembrolizumab, nivolumab, atezolizumab) and anti‐cytotoxic T‐lymphocyte antigen 4 (CTLA‐4) (ipilimumab), alone or in combination. Targeted therapies included BRAFi (i.e., vemurafenib, dabrafenib, encorafenib) alone or in combination with MEKi (binimetinib, cobimetinib, trametinib). All patients were in mutually exclusive treatment groups based on a treatment hierarchy rule: evidence of any SRS; no SRS, any WBRT; no SRS or WBRT, any systemic therapy; and no SRS, WBRT, or systemic therapy, or evidence of any other therapy. Additional analyses were also performed to evaluate outcomes according to specific treatment groups defined by radiation therapy (SRS and/or WBRT) and/or ICIs.

### 2.3 Outcomes and statistical analyses

Data were analyzed by period of CNS metastases diagnosis (2011–2014 vs 2015+), reflecting different therapy access eras. During 2011–2014, systemic therapies comprised BRAFi monotherapy and anti‐CTLA‐4 (ipilimumab), whereas 2015+ reflects approval and broader availability of BRAFi + MEKi combinations and anti‐PD‐(L)1 therapy alone or in combination with ipilimumab. Data were also analyzed by number of CNS metastases (≤3 vs >3), reflecting CNS tumor burden.

The primary outcome was OS (i.e., time from diagnosis of CNS metastasis until death or last contact, estimated using the Kaplan–Meier method). Multivariable analyses were performed using Cox proportional hazards modelling to evaluate the impact of specified covariates on risk of death. The model included factors identified from descriptive comparisons (initial treatment within 90 days of CNS metastatic diagnosis, CNS metastatic burden, and synchronous vs metachronous CNS metastatic diagnosis) as well as established prognostic factors for which sufficient data were available from charts (sex, race, presence of liver metastases, and extracranial metastatic burden). Additionally, interaction testing was performed to examine potential synergy between SRS/WBRT and ICIs.

## 3 RESULTS

### 3.1 Patients

Of 791 patients with melanoma and CNS metastases who had any encounter with the Flatiron Health network ≤90 days of metastatic diagnosis date, 392 (49.6%) had ≤3 and 383 (48.4%) had >3 CNS metastases (Table 1). Sixteen patients had an unknown number of CNS metastases and were excluded from analyses by CNS tumor burden. Most patients were from community centers (726/791; 91.8%) versus academic centers (65/791; 8.2%). More than half of patients (430/791; 54.4%) were aged ≥65 years, with a higher proportion of patients aged ≥65 years among those with >3 (222/383; 58.0%) versus ≤3 (200/392; 51.0%) CNS metastases. *BRAF* mutation‐positive tumors were more prevalent in patients with >3 (187/383; 48.8%) versus ≤3 (152/392; 38.8%) CNS metastases. Extracranial metastases were present in 672/791 (85.0%) and more prevalent in patients with >3 (345/383; 90.1%) versus ≤3 CNS metastases (312/392; 79.6%). Median number of involved extracranial sites was two (interquartile range [IQR], 1–4) and 2 (IQR, 1–3) in patients with >3 and ≤3 CNS metastases, respectively. Disease stage at initial diagnosis was I/II (185/791; 23.4%), III (133/791; 16.8%), IV (267/791; 33.8%), or not documented (206/791; 26.0%), and similar between cohorts. Median time from initial melanoma diagnosis to diagnosis of metastatic disease was 14.4 months (IQR, 0–40.9). Other baseline characteristics were similar between cohorts (Table 1; Table S1).

**TABLE 1:** Patient demographics and disease characteristics

Characteristic	Patients with CNS metastases (*N* = 791)	Number of CNS metastases^d^	
		≤3 (*n* = 392)	>3 (*n* =383)
Age at metastatic diagnosis (mean, SD)	63.9 (13.6)	63.1 (13.7)	64.9 (13.3)
Age group at metastatic diagnosis, *n* (%)			
<65 years	361 (45.6)	192 (49.0)	161 (42.0)
≥65 years	430 (54.4)	200 (51.0)	222 (58.0)
Sex, *n* (%)			
Female	244 (30.9)	127 (32.4)	110 (28.7)
Male	547 (69.2)	265 (67.6)	273 (71.3)
Race, *n* (%)			
White	636 (80.4)	315 (80.4)	310 (80.9)
Nonwhite	155 (19.6)	77 (19.6)	73 (19.1)
Asian	0 (0)	0 (0)	0 (0)
Black/African American	4 (0.5)	1 (0.3)	3 (0.8)
Hispanic/Latino	21 (2.7)	10 (2.6)	10 (2.6)
Other race	50 (6.3)	28 (7.1)	21 (5.5)
Unknown	80 (10.1)	38 (9.7)	39 (10.2)
Synchronous CNS metastases^a^			
No	269 (34.0)	128 (32.7)	133 (34.7)
Yes	522 (66.0)	264 (67.4)	250 (65.3)
Died	538 (68.0)	233 (59.4)	295 (77.0)
*BRAF* mutational status, *n* (%)			
Negative	343 (43.4)	198 (50.5)	138 (36.0)
Positive	347 (43.9)	152 (38.8)	187 (48.8)
Unknown/indeterminate	101 (12.8)	42 (10.7)	58 (15.1)
LDH level, *n* (%)^b^			
Unknown	314 (39.7)	162 (41.3)	146 (38.1)
<250 U/L	343 (43.4)	171 (43.6)	162 (42.3)
≥250 U/L	134 (16.9)	59 (15.1)	75 (19.6)
Extracranial metastases, *n* (%)	672 (85.0)	312 (79.6)	345 (90.1)
Number of extracranial sites involved, median (IQR)^c^	2 (1–3)	2 (1–3)	2 (1–4)
Sites of extracranial metastases, *n* (%)			
Lung	503 (63.6)	231 (58.9)	259 (67.6)
Other	291 (36.8)	133 (33.9)	154 (40.2)
Distant lymph node	236 (29.8)	108 (27.6)	124 (32.4)
Liver	223 (28.2)	89 (22.7)	126 (32.9)
Bone	210 (26.6)	76 (19.4)	128 (33.4)
Soft tissue	169 (21.4)	73 (18.6)	94 (24.5)
Skin	157 (19.9)	64 (16.3)	90 (23.5)

Abbreviations: CNS, central nervous system; IQR, interquartile range; LDH, lactate dehydrogenase.

^a^ Diagnosis of CNS metastasis ≤30 days of metastatic diagnosis.

^b^ Collected ≤60 days before or 30 days after metastasis diagnosis date.

^c^ 0 indicates isolated intracranial metastasis.

^d^ Patients with unknown number of metastases were not included due to the small sample size (*n* = 16).

Central nervous system metastases occurred early in most patients and survival outcomes remained stable, with shorter survival with higher intracranial tumor burden. Among the 791 patients with confirmed CNS metastases, synchronous CNS metastasis (≤30 days after first metastatic diagnosis) occurred in 522/791 patients (66.0%) and metachronous CNS metastasis (>30 days after first metastatic diagnosis) occurred in 269/791 patients (34.0%). Median time from CNS metastatic diagnosis to death or last contact was 4.9 months (IQR, 2.0–11.0), and was longer in patients with ≤3 versus >3 CNS metastases (7.0 months [IQR, 3.0–16.0] vs 3.0 months [IQR, 1.9–6.5]) but similar between 2011 and 2014 versus 2015+ (5.0 months [IQR, 2.0–12] vs 4.4 months [IQR, 2.0–10.3]).

### 3.2 Treatment patterns

Stereotactic radiosurgery was the most common treatment ≤90 days after CNS metastatic diagnosis (320/791; 40.5%) and more frequent in 2015+ (201/456; 44.1%) versus 2011–2014 (119/335; 35.5%; Table 2). Use of SRS was more frequent in patients with ≤3 (232/392; 59.2%) versus >3 CNS metastases (85/383; 22.2%). For patients with >3 CNS metastases, the most common initial treatment was WBRT (152/383; 39.7%). There was no evidence of treatment (systemic or local) ≤90 days of CNS metastatic diagnosis for 155/791 patients (19.6%).

**TABLE 2:** First treatment^a^ received ≤90 days after CNS metastasis diagnosis

Treatment, *n* (%)	Patients with CNS metastases (*N* = 791)	CNS metastasis diagnosis year		Number of CNS metastases	
		2011–2014 (*n* = 335)	2015+ (*n* = 456)	≤3 (*n* = 392)	>3 (*n* = 383)
No evidence of treatment	155 (19.6)	75 (22.4)	80 (17.5)	72 (18.4)	79 (20.6)
Any SRS	320 (40.5)	119 (35.5)	201 (44.1)	232 (59.2)	85 (22.2)
No SRS, any WBRT	195 (24.7)	95 (28.4)	100 (21.9)	40 (10.2)	152 (39.7)
No SRS/WBRT, any systemic	90 (11.4)	31 (9.3)	59 (12.9)	28 (7.1)	60 (15.7)
Other therapies	31 (3.9)	15 (4.5)	16 (3.5)	20 (5.1)	7 (1.8)

Abbreviations: CNS, central nervous system; SRS, stereotactic radiosurgery; WBRT, whole‐brain radiation therapy.

^a^ Mutually exclusive groups based on treatment hierarchy rule.

Immune checkpoint inhibitors were the most common systemic therapy used in combination with defined local therapies. Across treatment groups by treatment hierarchy rule, ICI use increased from 18.2% in 2011–2014 (anti‐PD‐[L]1, 1.2%; anti‐CTLA‐4, 17.0%; anti‐PD‐[L]1 + anti‐CTLA‐4, 0.0%) to 39.5% in 2015+ (anti‐PD‐[L]1, 20.6%; anti‐CTLA‐4, 3.7%; anti‐PD‐[L]1 + anti‐CTLA‐4, 15.1%; Table 3). In patients with initial SRS treatment, ICI use increased (24.4% to 45.3%); anti‐CTLA‐4 use decreased (22.7% to 5.5%), whereas anti‐PD‐(L)1 use increased (1.7% to 21.9%) and anti–PD‐(L)1 + anti‐CTLA‐4 increased (0.0% to 17.9%).

**TABLE 3:** Specific first therapy ≤90 days after CNS metastasis diagnosis^a^

Treatment, *n* (%)	2011–2014 (*n* = 335)				2015+ (*n* = 456)			
	Any SRS (*n* = 119)	No SRS, any WBRT (*n* = 95)	No SRS/WBRT, any systemic (*n* = 31)	Other therapies (*n* = 15)	Any SRS (*n* = 201)	No SRS, any WBRT (*n* = 100)	No SRS/WBRT, any systemic (*n* = 59)	Other therapies (*n* = 16)
ICIs	29 (24.4)	17 (17.9)	15 (48.4)	—	91 (45.3)	48 (48.0)	41 (69.5)	—
Anti‐PD‐(L)1	2 (1.7)	1 (1.1)	1 (3.2)	—	44 (21.9)	26 (26.0)	24 (40.7)	—
Anti‐CTLA‐4	27 (22.7)	16 (16.8)	14 (45.2)	—	11 (5.5)	3 (3.0)	3 (5.1)	—
Anti‐PD‐ (L)1 + anti‐CTLA‐4	—	—	—	—	36 (17.9)	19 (19.0)	14 (23.7)	—
Targeted therapy	23 (19.3)	15 (15.8)	14 (45.2)	—	31 (15.4)	10 (10.0)	24 (40.7)	—
BRAFi	23 (19.3)	15 (15.8)	14 (45.2)	—	22 (10.9)	8 (8.0)	21 (35.6)	—
BRAFi + MEKi	—	—	—	—	9 (4.5)	2 (2.0)	3 (5.1)	—
Local								
SRS	119 (100)	—	—	—	201 (100)	—	—	—
WBRT	20 (16.8)	95 (100)	—	—	13 (6.5)	100 (100)	—	—
Craniotomy	23 (19.3)	9 (9.5)	4 (12.9)	11 (73.3)	51 (25.4)	16 (16.0)	4 (6.8)	13 (81.3)

Abbreviations: BRAFi, BRAF inhibitor; CNS, central nervous system; CTLA‐4, cytotoxic T‐lymphocyte antigen 4; ICI, immune checkpoint inhibitor; MEKi, MEK inhibitor; PD‐(L)1, programmed death ligand‐1; SRS, stereotactic radiosurgery; WBRT, whole‐brain radiation therapy.

^a^ Mutually exclusive groups based on treatment hierarchy rule.

Targeted therapy use remained stable from 2011–2014 (15.5%) to 2015+ (14.3%). Although BRAFi + MEKi combination therapy increased (0.0% to 3.1%), BRAFi monotherapy was the most common targeted therapy (15.5% [2011–2014]; 11.2% [2015+]) (Table 3). In patients with initial SRS treatment, BRAFi + MEKi therapy increased (0.0% to 4.5%), whereas BRAFi monotherapy decreased (19.3%–11.0%). Median time from first metastatic diagnosis to initiation of first systemic treatment was 36 days (range, 0–90 days).

### 3.3 Survival

**FIGURE 1:**
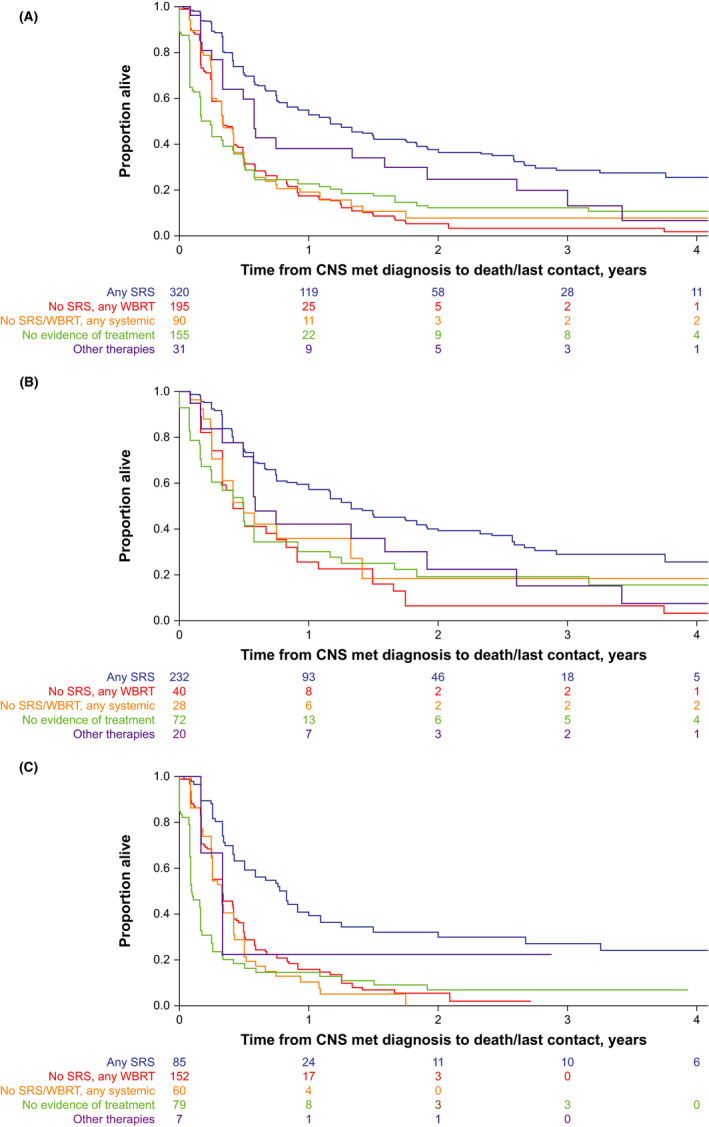
Kaplan–Meier curves of overall survival by first treatment^a^ received ≤90 days of diagnosis of CNS metastasis in (A) all patients with CNS metastases, (B) patients with ≤3 CNS metastases, and (C) patients with >3 CNS metastases. ^a^Mutually exclusive groups based on treatment hierarchy rule. Abbreviations: CNS, central nervous system; met, metastasis; SRS, stereotactic radiosurgery; WBRT, whole‐brain radiation therapy

Median OS among all patients was longest in patients who received initial SRS treatment (1.17 years [95% CI, 0.91–1.50]) and shortest with no evidence of treatment (0.25 years [95% CI, 0.17–0.33]; Figure 1A; Table 4). Among SRS‐treated patients, 1‐ and 2‐year OS rates were 52.7% (95% CI, 46.5–58.6) and 37.2% (95% CI, 30.8–43.6), respectively (Figure 1A; Table 4). Among patients with no evidence of treatment, 1‐ and 2‐year OS rates were 22.6% (95% CI, 15.6–30.5) and 12.1% (95% CI, 6.6–19.5), respectively (Figure 1B; Table 4).

**TABLE 4:** Overall survival by first treatment^a^ received ≤90 days after CNS metastasis diagnosis

Overall survival	Patients with CNS metastases (*N* = 791)	CNS metastasis diagnosis year		Number of CNS metastases	
		2011–2014 (*n* = 335)	2015+ (*n* = 456)	≤3 (*n* = 392)	>3 (*n* = 383)
Any SRS	(*n* = 320)	(*n* = 119)	(*n* = 201)	(*n* = 232)	(*n* = 85)
Median OS	1.17 (0.91–1.50)	0.83 (0.75–1.17)	1.75 (1.17–2.42)	1.33 (1.00–1.83)	0.83 (0.50–1.08)
1‐year OS	52.7 (46.5–58.6)	43.6 (33.9–52.9)	59.0 (51.1–66.1)	57.1 (49.7–63.8)	39.3 (27.9–50.5)
2‐year OS	37.2 (30.8–43.6)	27.9 (19.5–37.0)	44.0 (35.0–52.7)	40.0 (32.3–47.6)	29.5 (18.6–41.2)
No SRS, any WBRT	(*n* = 195)	(*n* = 95)	(*n* = 100)	(*n* = 40)	(*n* = 152)
Median OS	0.34 (0.33–0.42)	0.34 (0.25–0.42)	0.33 (0.25–0.45)	0.42 (0.33–0.83)	0.33 (0.25–0.42)
1‐year OS	17.4 (12.0–23.7)	18.0 (10.7–26.7)	16.6 (9.1–26.1)	25.6 (12.7–40.6)	15.5 (9.7–22.5)
2‐year OS	5.2 (2.3–10.0)	6.0 (2.2–12.5)	5.6 (1.5–13.5)	6.4 (1.2–18.3)	5.4 (2.1–11.1)
No SRS/WBRT, any systemic	(*n* = 90)	(*n* = 31)	(*n* = 59)	(*n* = 28)	(*n* = 60)
Median OS	0.33 (0.25–0.42)	0.33 (0.25–0.50)	0.34 (0.25–0.50)	0.50 (0.25–1.33)	0.33 (0.25–0.41)
1‐year OS	19.1 (11.0–29.0)	12.1 (3.5–26.5)	24.4 (12.7–38.0)	36.2 (16.7–56.2)	10.3 (3.7–20.8)
2‐year OS	7.8 (2.4–17.6)	8.1 (1.6–21.8)	5.3 (0.5–20.4)	18.1 (3.6–41.6)	0 (0–0)
No evidence of treatment	(*n* = 155)	(*n* = 75)	(*n* = 80)	(*n* = 72)	(*n* = 79)
Median OS	0.25 (0.17–0.33)	0.25 (0.17–0.50)	0.17 (0.08–0.33)	0.50 (0.25–0.58)	0.09 (0.08–0.17)
1‐year OS	22.6 (15.6–30.5)	29.5 (19.3–40.3)	13.4 (5.5–25.0)	30.0 (18.7–42.1)	14.5 (6.9–24.8)
2‐year OS	12.1 (6.6–19.5)	15.7 (8.0–25.9)	6.7 (0.8–22.1)	19.0 (9.2–31.6)	6.8 (2.0–15.7)
Other therapies	(*n* = 31)	(*n* = 15)	(*n* = 16)	(*n* = 20)	(*n* = 7)
Median OS	0.58 (0.33–1.59)	0.59 (0.17–1.91)	0.54 (0.17–3.00)	0.59 (0.50–1.91)	0.33 (0.17‐NE)
1‐year OS	38.4 (19.7–57.0)	34.9 (11.0–60.6)	41.7 (15.3–66.5)	41.8 (19.0–63.3)	22.2 (1.0–61.5)
2‐year OS	24.9 (9.8–43.5)	13.1 (0.9–41.2)	33.3 (10.3–58.8)	22.4 (6.2–44.6)	22.2 (1.0–61.5)

Note

All data presented are years, % (95% CI).

Abbreviations: CNS, central nervous system; NE, not estimable; OS, overall survival; SRS, stereotactic radiosurgery; WBRT, whole‐brain radiation therapy.

^a^ Mutually exclusive groups based on treatment hierarchy rule.

Among patients treated with radiation therapy (SRS and/or WBRT), median OS was longest for patients who received initial treatment with SRS in combination with ICIs (1.92 years [95% CI, 1.25–2.58]) followed by those who received SRS alone (1.51 years [95% CI, 0.75–2.59]; Figure S2; Table S2). Among patients treated with SRS and ICIs, 1‐ and 2‐year OS rates were 63.1% (95% CI, 52.5–72.0) and 46.2% (95% CI, 34.4–57.2), respectively; among those treated with SRS alone, 1‐ and 2‐year OS rates were 58.3% (95% CI, 45.7–69.0) and 44.7% (95% CI, 31.8–56.8), respectively (Figure S2; Table S2). Median OS for patients treated with ICIs alone was 0.33 years (95% CI, 0.25–0.42), with 1‐ and 2‐year OS rates of 16.6% (95% CI, 7.4–28.9) and 12.4% (95% CI, 4.2–25.4), respectively (Figure S2; Table S2).

Stereotactic radiosurgery was associated with improved OS outcomes regardless of intracranial tumor burden; OS for patients with high intracranial tumor burden was shorter than for low intracranial tumor burden. Patients treated with SRS had the longest median OS for low (≤3 CNS metastases: 1.33 years [95% CI, 1.00–1.83]) and high intracranial tumor burden (>3 CNS metastases: 0.83 years [95% CI, 0.50–1.08]; Figure 1B,C). Among SRS‐treated patients, 1‐year OS rates were 57.1% (95% CI, 49.7–63.8) and 39.3% (95% CI, 27.9–50.5) in patients with ≤3 and >3 CNS metastases, and 2‐year OS rates were 40.0% (95% CI, 32.3–47.6) and 29.5% (95% CI, 18.6–41.2), respectively (Figure 1B,C; Table 4).

**FIGURE 2:**
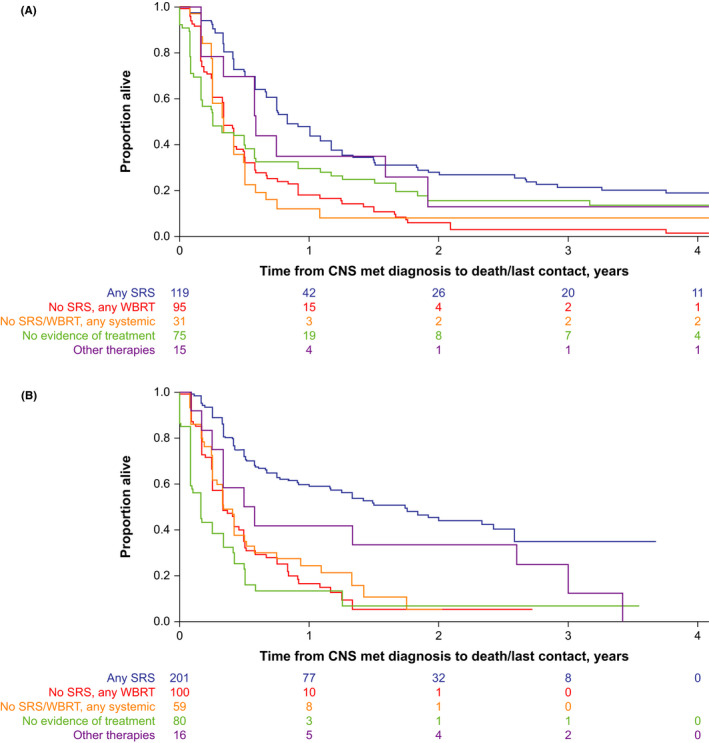
Kaplan–Meier curves of overall survival by first treatment^a^ received ≤90 days of diagnosis of CNS metastasis in (A) patients diagnosed with CNS metastasis from 2011 to 2014, and (B) patients diagnosed with CNS metastasis from 2015 or later. ^a^Mutually exclusive groups based on treatment hierarchy rule. Abbreviations: CNS, central nervous system; met, metastasis; SRS, stereotactic radiosurgery; WBRT, whole‐brain radiation therapy

Survival outcomes for patients treated with SRS improved over time. Median OS for patients receiving any SRS treatment increased from 0.83 years (95% CI, 0.75–1.17; 2011–2014) to 1.75 years (95% CI, 1.17–2.42; 2015+; Figure 2A,B; Table 4). One‐year OS rates were 43.6% (95% CI, 33.9–52.9) and 59.0% (95% CI, 51.1–66.1) in 2011–2014 and 2015+, and 2‐year OS rates were 27.9% (95% CI, 19.5–37.0) and 44.0% (95% CI, 35.0–52.7), respectively (Figure 2A,B; Table 4). Patients with no evidence of treatment had a median OS of 0.25 (95% CI, 0.17–0.50) and 0.17 (95% CI, 0.08–0.33) years in 2011–2014 and 2015+ (Figure 2A,B), with 1‐year OS rates of 29.5% (95% CI, 19.3–40.3) and 13.4% (95% CI, 5.5–25.0) and 2‐year OS rates of 15.7% (95% CI, 8.0–25.9) and 6.7% (95% CI, 0.8–22.1), respectively.

Synchronous CNS metastasis was associated with longer median OS (0.58 years [95% CI, 0.50–0.67]) compared with metachronous CNS metastasis (0.42 years [95% CI, 0.33–0.50]; Figure S3). Median OS was longer for patients with synchronous versus metachronous CNS metastasis among those with ≤3 CNS metastases (1.17 years [95% CI, 0.75–1.49] vs 0.58 years [95% CI, 0.42–0.75], respectively), but not in those with >3 CNS metastases (0.33 years [95% CI, 0.33–0.42] vs 0.29 years [95% CI, 0.25–0.41], respectively; Figure S3).

In the multivariable Cox proportional hazards model, any ICI treatment (vs other/no evidence of treatment, hazard ratio 0.74 [95% CI, 0.60–0.92]; *p* = 0.01), SRS treatment (vs no SRS or WBRT, hazard ratio 0.45 [95% CI, 0.36–0.55]; *p* < 0.0001) and synchronous CNS metastasis (vs metachronous CNS metastasis, hazard ratio 0.81 [95% CI, 0.67–0.97]; *p* = 0.02) were independently associated with significantly reduced risk of death (Table 5). Presence of >3 CNS metastases (vs ≤3, hazard ratio 1.77 [95% CI, 1.46–2.14]; *p* < 0.0001) and presence of liver metastases (vs no liver metastases, hazard ratio 1.23 [95% CI, 1.00–1.51]; *p* = 0.05) were independently associated with significantly increased risk of death (Table 5). Number of extracranial metastatic sites (≥3 vs <3) was not independently associated with risk of death (hazard ratio 1.09 [95% CI, 0.90–1.33]; *p* = 0.38). Interaction testing demonstrated no significant interaction between SRS/WBRT and ICIs (*p* = 0.12) or between SRS and ICIs (*p* = 0.94).

**TABLE 5:** Cox proportional hazards model for risk of death

	Hazard ratio	95% CI		*p* value
Systemic treatment ≤90 days of diagnosis of CNS metastasis (vs other/no evidence of treatment)				
Any ICIs	0.74	0.60	0.92	0.01
Any targeted therapy	0.82	0.63	1.06	0.13
Any chemotherapy	1.21	0.76	1.95	0.43
SRS/WBRT ≤90 days of diagnosis of CNS metastasis (vs no SRS or WBRT)				
Any SRS	0.45	0.36	0.55	<0.0001
No SRS, any WBRT	0.96	0.77	1.19	0.69
Female (vs male)	0.87	0.73	1.05	0.16
Nonwhite race (vs White)	1.11	0.34	0.89	0.34
Number of CNS metastases (vs ≤3)				
>3	1.77	1.46	2.14	<0.0001
Unknown	0.76	0.40	1.45	0.40
Presence of liver metastases (vs no liver metastases)	1.23	1.00	1.51	0.05
Extracranial metastatic sites ≥3 (vs <3)	1.09	0.90	1.33	0.38
Synchronous CNS metastasis (vs metachronous CNS metastasis)^a^	0.81	0.67	0.97	0.02

Abbreviations: CNS, central nervous system; SRS, stereotactic radiosurgery; WBRT, whole‐brain radiation therapy.

^a^ Synchronous CNS metastasis = diagnosis of CNS metastasis ≤30 days of metastatic diagnosis; Metachronous CNS metastasis = diagnosis of CNS metastasis >30 days after metastatic diagnosis.

## 4 DISCUSSION

Real‐world data from US patients suggest that clinical outcomes for patients with melanoma and CNS metastases remain poor, particularly for patients with higher intracranial tumor burden. Initial SRS treatment had the most favorable survival outcomes and increased in prevalence over time. Median OS for patients treated with SRS improved in 2015+ versus 2011–2014, which may reflect improvements in technology, administration, and availability. Patients who are candidates for SRS have lower number of metastases and smaller tumor burden, and are likely to have better OS. There was increased use of combination ICIs, and previous reports have suggested potential synergistic interaction between SRS and immunotherapy or targeted therapy, which may improve OS.^20^, ^27^, ^28^, ^29^ Use of anti‐PD‐(L)1 alone and in combination with anti‐CTLA‐4 increased from 1.7% to 21.9% and 0.0% to 17.9%, and use of BRAFi + MEKi combination therapy increased from 0.0% to 4.5% from 2011–2014 to 2015+ among SRS‐treated patients, respectively. While use of WBRT and BRAFi monotherapy decreased slightly from 2011–2014 to 2015+, it remained higher than expected, suggesting a degree of sub‐optimal treatment in the real‐world setting.

Patients with no evidence of treatment had the least favorable survival outcomes; median OS (3 months) was similar to untreated patients with melanoma and CNS metastases in the SEER‐Medicare database (2.1 months), indicating that patients who were not candidates for a therapeutic intervention were likely close to end of life.^30^ Under current guidelines, WBRT may be appropriate in the palliative setting when SRS is not feasible and patients have failed systemic therapy. Median OS in patients with no SRS, any WBRT was 4.1 months, irrespective of time period, and was consistent with SEER‐Medicare WBRT OS (2.7 months).^30^ These data highlight the limited OS benefit with WBRT. For patients who received no SRS/WBRT and any systemic therapy, 1‐year OS rates increased from 12.1% (2011–2014) to 24.4% (2015+). However, 2‐year OS rates were similar; long‐term outcomes may reflect, in part, limited activity and/or durability of response with current systemic therapies in patients with symptomatic CNS metastases, although the use of combination ICIs, which have the highest rate of durable responses, was too limited to assess impact.^18^, ^19^, ^21^, ^31^

Additional population‐based cohort studies of patients with melanoma and CNS metastases demonstrated similar changes in treatment patterns over time. A Canadian study demonstrated increased use of conformal radiotherapy (including SRS) and immunotherapy, and improvements in OS, with 1‐year OS rates increasing from 12.3% (2007–2009) and 10.7% (2010–2012) to 21.8% (2013–2016).^32^ Further comparisons between our study and the Canadian study are limited due to treatment category and time period differences. An additional report from Australia from January 2011 through December 2014 demonstrated that patients who received SRS combined with systemic therapy had improved outcomes compared with other therapies, including SRS alone.^33^ In our study, patients who received initial treatment with SRS combined with ICIs had slightly better OS than those who received SRS alone. Both SRS and ICIs were independently associated with improved OS outcomes in multivariable analysis. However, interaction testing to examine potential synergy between SRS/WBRT and ICIs did not demonstrate a significant effect.

In the current analysis, synchronous CNS metastasis diagnosis was associated with improved survival outcomes for patients with <3 CNS metastases. This may support routine CNS imaging for early detection and treatment of CNS metastases, especially in those with low intracranial burden. In the Canadian study, use of brain magnetic resonance imaging for surveillance or metastatic restaging increased over time and was associated with better survival outcomes in both patient‐level and regional‐level analyses, suggestive of a survival benefit with earlier CNS metastases detection.^32^

Our analysis provides valuable insight into real‐world clinical practice with longitudinal evaluation reflecting recent advances in melanoma therapy. However, we acknowledge several limitations. Most patients were treated at community rather than academic practices and do not reflect potential differences in patient populations and treatment patterns between these settings. Electronic health record databases, while less costly and time consuming compared to primary data collection, may contain missing or incomplete data. Patients may have received noncaptured treatment outside of the Flatiron Health network. Additionally, the absence of links to claims data prevented reliable capture of information on use of oral medications such as corticosteroids that may influence survival outcomes. Based on validated algorithms developed for the prediction of benefit from SRS and selection of eligible patients, intracranial tumor burden was categorized as ≤3 versus >3 CNS metastases. Abstraction methodology limitations prevented a more granular assessment of the impact of CNS tumor burden, particularly for patients with >3 CNS metastases. Analyses were limited to treatments received ≤90 days after diagnosis and did not consider additional treatments received following this time.

## 5 CONCLUSION

Despite expansion of effective therapies for patients with metastatic melanoma, patients with CNS metastases, particularly with greater tumor burden, continue to have poor survival outcomes, representing an area of high unmet need. Real‐world data from the current study highlight the increased prevalence of SRS over time and associated improved outcomes, potentially as it is used more consistently along with ICIs. The benefit of concurrent SRS and ICIs remains uncertain and requires confirmation in well‐controlled clinical studies.

## CONFLICT OF INTEREST

HT: Research support and/or personal fees from Bristol Myers Squibb, Merck, Genentech, Celgene, GlaxoSmithKline, Array, and Novartis. TMT, KB, NS, and EM: Employees and shareholders of Genentech/F. Hoffmann‐La Roche. EB and LH: No conflicts.

## AUTHOR CONTRIBUTIONS

HT, TMT, KB, NS, and EM: Conceptualization, investigation, and writing (initial draft, review, and editing). EB and LH: Conceptualization, investigation, and writing (review, editing).

## ETHICS STATEMENT

Institutional review board approval of the study protocol with a waiver of informed consent was obtained prior to study conduct.

## Supporting information

Supplementary Material

## Data Availability

Qualified researchers may request access to individual patient‐level data through the clinical study data request platform (www.clinicalstudydatarequest.com). Further details on Roche's criteria for eligible studies are available here (https://clinicalstudydatarequest.com/Study‐Sponsors/Study‐Sponsors‐Roche.aspx). For further details on Roche's Global Policy on the Sharing of Clinical Information and how to request access to related clinical study documents, see here (https://www.roche.com/research_and_development/who_we_are_how_we_work/clinical_trials/our_commitment_to_data_sharing.htm).
