# Supplementary material for: Treatment patterns and clinical outcomes for patients with melanoma and central nervous system metastases: A real‐world study
Source: Cancer Med. 2021 Dec 7;11(1):139–50. doi: 10.1002/cam4.4438 (PMC8704162; doi:10.1002/cam4.4438)
Supplement: Supplementary file 1 — Supplementary Material [file CAM4-11-139-s001.docx]

**Supplemental Figures**

**Supplemental Figure S1.** Study design and attrition. ^a^May be concurrent with diagnosis of advanced disease. ^b^May be concurrent with first diagnosis of metastatic disease.


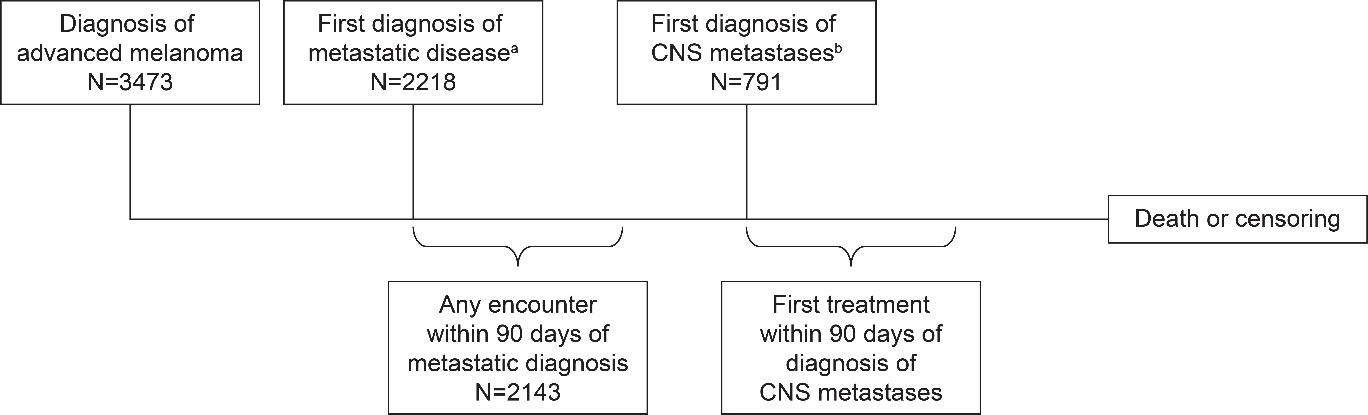


**Supplemental Figure S2.** Kaplan-Meier curves of overall survival by specific first therapy (radiation therapy and/or ICIs)^a^ ≤90 days of diagnosis of CNS metastasis**.**

^a^No patients received WBRT alone.

Abbreviations: CNS, central nervous system; met, metastasis; SRS, stereotactic radiosurgery; WBRT, whole-brain radiation therapy.

**
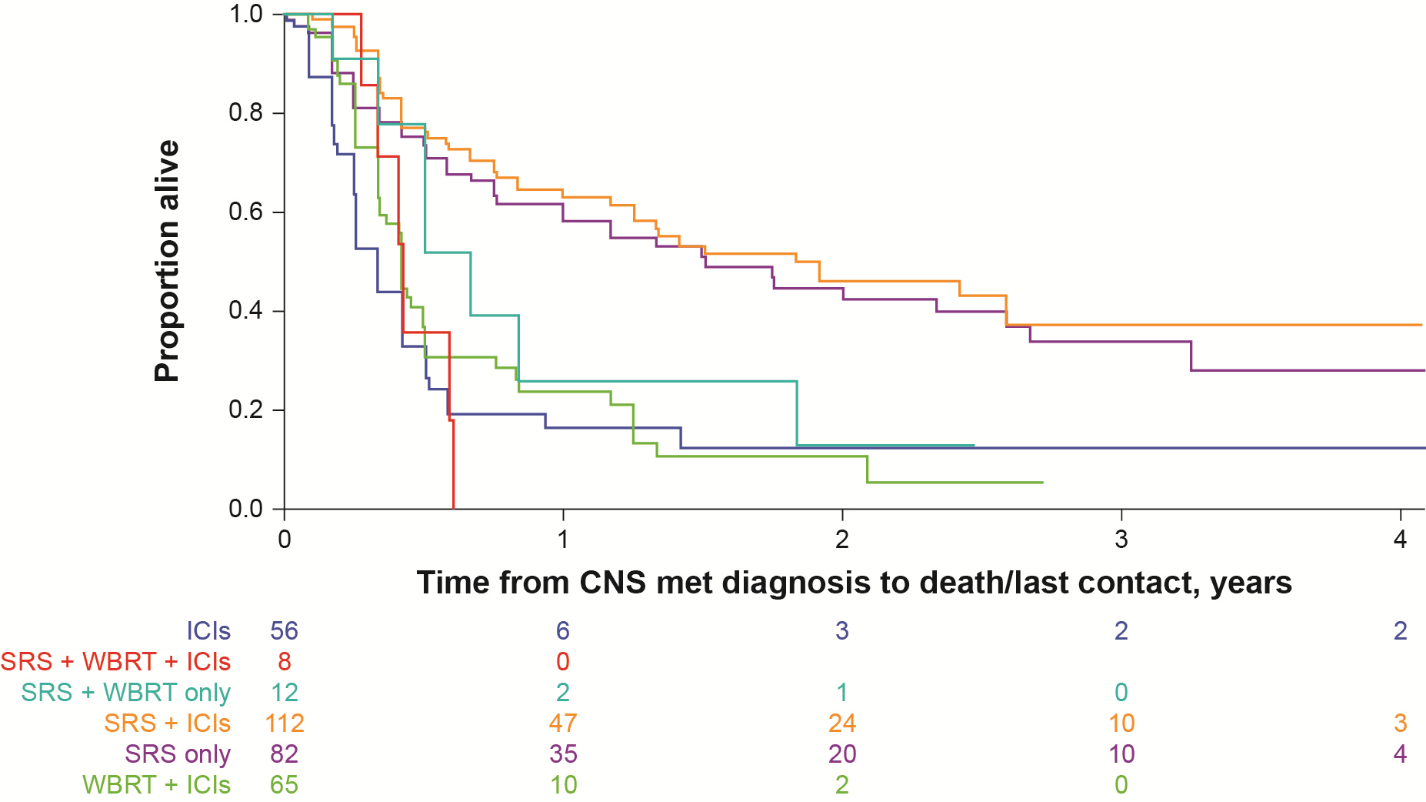
**

**Supplemental Figure S3.** Kaplan-Meier curves of overall survival by synchronous or metachronous CNS metastasis^a^ in (a) all patients with CNS metastases, (b) patients with ≤3 CNS metastases, and (c) patients with >3 CNS metastases. ^a^Synchronous CNS metastasis was defined as diagnosis of CNS metastases ≤30 days of metastatic diagnosis; metachronous CNS metastasis was defined as diagnosis of CNS metastases >30 days after metastatic diagnosis. Abbreviations: Mets, metastasis

**
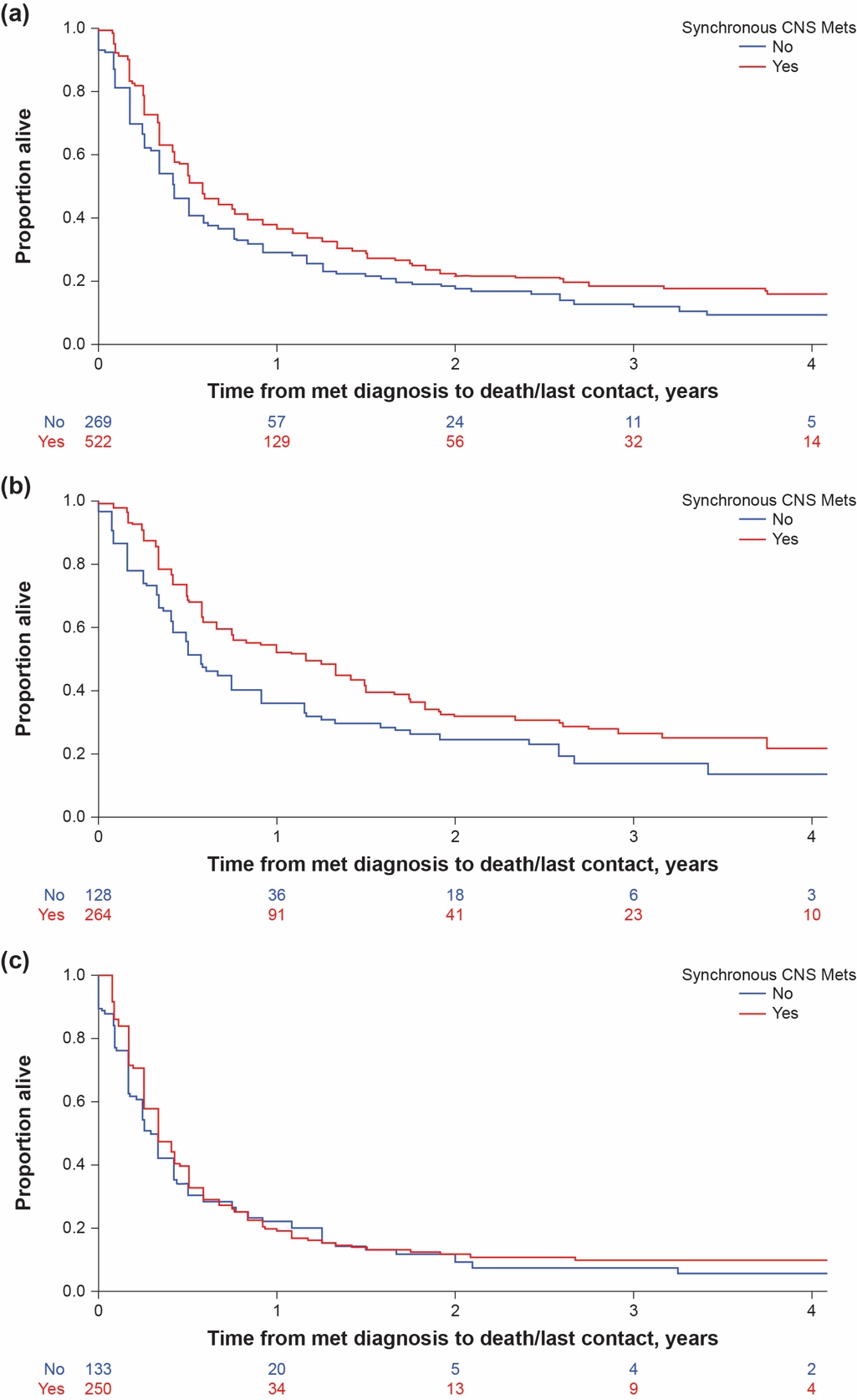
**

**Supplemental Table S1.** Patient Demographics and Disease Characteristics by Treatment Cohort

| **Characteristic** | **Any SRS**  **(n=320)** | **No SRS, Any WBRT (n=195)** | **No SRS/WBRT, any systemic (n=90)** | **Other therapies (n=31)** | **No Evidence of treatment**  **(n=155)** |
| --- | --- | --- | --- | --- | --- |
| Age at metastatic diagnosis (mean, SD) | 62.7 (13.4) | 64.7 (13.3) | 63.0 (12.4) | 65.0 (15.0) | 65.7 (14.7) |
| Age group at metastatic diagnosis, n (%) |  |  |  |  |  |
| <65 years | 156 (48.8) | 85 (43.6) | 45 (50.0) | 14 (45.2) | 61 (39.4) |
| ≥65 years | 164 (51.3) | 110 (56.4) | 45 (50.0) | 17 (54.8) | 94 (60.6) |
| Sex, n (%) |  |  |  |  |  |
| Female | 107 (33.4) | 57 (29.9) | 31 (34.4) | 11 (35.5) | 38 (24.5) |
| Male | 213 (66.6) | 138 (70.8) | 59 (65.5) | 20 (64.5) | 117 (75.5) |
| Race, n (%) |  |  |  |  |  |
| White | 259 (80.9) | 161 (82.6) | 70 (77.8) | 24 (77.4) | 122 (78.7) |
| Nonwhite | 61 (19.1) | 34 (17.4) | 20 (22.2) | 7 (22.6) | 33 (21.3) |
| Asian | 0 (0) | 0 (0) | 0 (0) | 0 (0) | 0 (0) |
| Black/African American | 1 (0.3) | 1 (0.5) | 1 (1.1) | 0 (0) | 1 (0.6) |
| Hispanic/Latino | 7 (2.2) | 5 (2.6) | 2 (2.2) | 1 (3.2) | 6 (3.9) |
| Other race | 28 (8.8) | 9 (4.6) | 5 (5.6) | 3 (9.7) | 5 (3.2) |
| Unknown | 25 (7.8) | 19 (9.7) | 12 (13.3) | 3 (9.7) | 21 (13.5) |
| Number of CNS metastases, n (%) |  |  |  |  |  |
| ≤3 | 232 (72.5) | 40 (20.5) | 28 (31.1) | 20 (64.5) | 72 (46.5) |
| >3 | 85 (26.6) | 152 (78.0) | 60 (66.7) | 7 (22.6) | 79 (51.0) |
| Unknown | 3 (0.9) | 3 (1.5) | 2 (2.2) | 4 (12.9) | 4 (2.6) |
| Synchronous CNS metastasis^a^ |  |  |  |  |  |
| No | 104 (32.5) | 68 (34.9) | 15 (16.7) | 14 (45.2) | 68 (43.9) |
| Yes | 216 (67.5) | 127 (65.1) | 75 (83.3) | 17 (54.8) | 87 (56.1) |
| Died | 172 (53.8) | 165 (84.6) | 68 (75.6) | 22 (71.0) | 111 (71.6) |
| *BRAF* mutational status, n (%) |  |  |  |  |  |
| Negative | 163 (50.9) | 76 (39.0) | 29 (32.2) | 15 (48.4) | 60 (38.7) |
| Positive | 137 (42.8) | 90 (46.2) | 56 (62.2) | 9 (29.0) | 55 (35.5) |
| Unknown/indeterminate | 20 (6.3) | 29 (14.9) | 5 (5.6) | 7 (22.6) | 40 (25.8) |
| LDH level, n (%)^b^ |  |  |  |  |  |
| Unknown | 127 (39.7) | 70 (35.9) | 30 (33.3) | 15 (48.4) | 72 (46.5) |
| <250 U/L | 142 (44.4) | 90 (46.2) | 36 (40.0) | 14 (45.2) | 61 (39.4) |
| ≥250 U/L | 51 (15.9) | 35 (17.9) | 24 (26.7) | 2 (6.5) | 22 (14.2) |
| Extracranial metastases, n (%) | 269 (84.1) | 170 (87.2) | 77 (85.6) | 28 (90.3) | 128 (82.6) |
| Number of extracranial sites involved, median (IQR)^c^ | 2 (1-4) | 2 (1-3) | 3 (1-4) | 2 (1-4) | 2 (1-3) |
| Sites of extracranial metastases, n (%) |  |  |  |  |  |
| Lung | 209 (65.3) | 118 (60.5) | 61 (67.8) | 25 (80.7) | 90 (58.1) |
| Other | 119 (37.2) | 73 (37.4) | 36 (40.0) | 10 (32.3) | 53 (34.2) |
| Distant lymph node | 100 (31.3) | 49 (25.1) | 30 (33.3) | 7 (22.6) | 50 (32.3) |
| Liver | 80 (25.0) | 63 (32.3) | 32 (35.6) | 11 (35.5) | 37 (23.9) |
| Bone | 72 (22.5) | 51 (26.2) | 40 (44.4) | 7 (22.6) | 40 (25.8) |
| Soft tissue | 71 (22.2) | 41 (21.0) | 20 (22.2) | 4 (12.9) | 33 (21.3) |
| Skin | 65 (20.3) | 47 (24.1) | 16 (17.8) | 6 (19.4) | 23 (14.8) |

^a^≤30 days of metastatic diagnosis.

^b^Collected ≤60 days before or 30 days after metastasis diagnosis date.

^c^0 indicates isolated intracranial metastasis.

^d^Patients with unknown number of metastases were not included due to the small sample size (n=16).
Abbreviations: CNS, central nervous system; IQR, interquartile range; LDH, lactate dehydrogenase.

**Supplemental Table S2.** Overall Survival by Specific First Therapy (Radiation Therapy and/or ICIs)^a^ ≤90 Days After CNS Metastasis Diagnosis

| **Overall survival** | **Patients with CNS metastases**  **(N=791)** |
| --- | --- |
| SRS + ICIs | (n=112) |
| Median OS | 1.92 (1.25-2.58) |
| 1-year OS | 63.1 (52.5-72.0) |
| 2-year OS | 46.2 (34.4-57.2) |
| SRS alone | (n=82) |
| Median OS | 1.51 (0.75-2.59) |
| 1-year OS | 58.3 (45.7-69.0) |
| 2-year OS | 44.7 (31.8-56.8) |
| SRS + WBRT | (n=12) |
| Median OS | 0.67 (0.33-1.83) |
| 1-year OS | 26.0 (3.9-57.2) |
| 2-year OS | 13.0 (0.68-43.4) |
| SRS + WBRT + ICIs | (n=8) |
| Median OS | 0.42 (0.27-NE) |
| 1-year OS | 0 (NE-NE) |
| 2-year OS | 0 (NE-NE) |
| WBRT + ICIs | (n=65) |
| Median OS | 0.42 (0.33-0.50) |
| 1-year OS | 23.9 (13.2-36.2) |
| 2-year OS | 10.6 (3.6-22.0) |
| ICIs alone | (n=56) |
| Median OS | 0.33 (0.25-0.42) |
| 1-year OS | 16.6 (7.4-28.9) |
| 2-year OS | 12.4 (4.2-25.4) |

All data presented are years, % (95% CI).

^a^No patients received WBRT alone.

Abbreviations: CNS, central nervous system; ICIs, immune checkpoint inhibitors; NE, not estimable; OS, overall survival; SRS, stereotactic radiosurgery; WBRT, whole-brain radiation therapy.
